# Machine Learning Model Insights into Base-Catalyzed Hydrothermal Lignin Depolymerization

**DOI:** 10.1021/acsomega.3c04168

**Published:** 2023-08-24

**Authors:** Abraham Castro Garcia, Shuo Cheng, Shawn E. McGlynn, Jeffrey S. Cross

**Affiliations:** † Department of Transdisciplinary Science and Engineering, School of Environment and Society, Tokyo Institute of Technology, 2-12-1 S6-10, Ookayama, Meguro-ku, Tokyo 152-8552, Japan; ‡ Earth-Life Science Institute, Tokyo Institute of Technology, Meguro, Tokyo 152-8550, Japan; § Blue Marble Space Institute of Science, Seattle, Washington 98101, United States

## Abstract

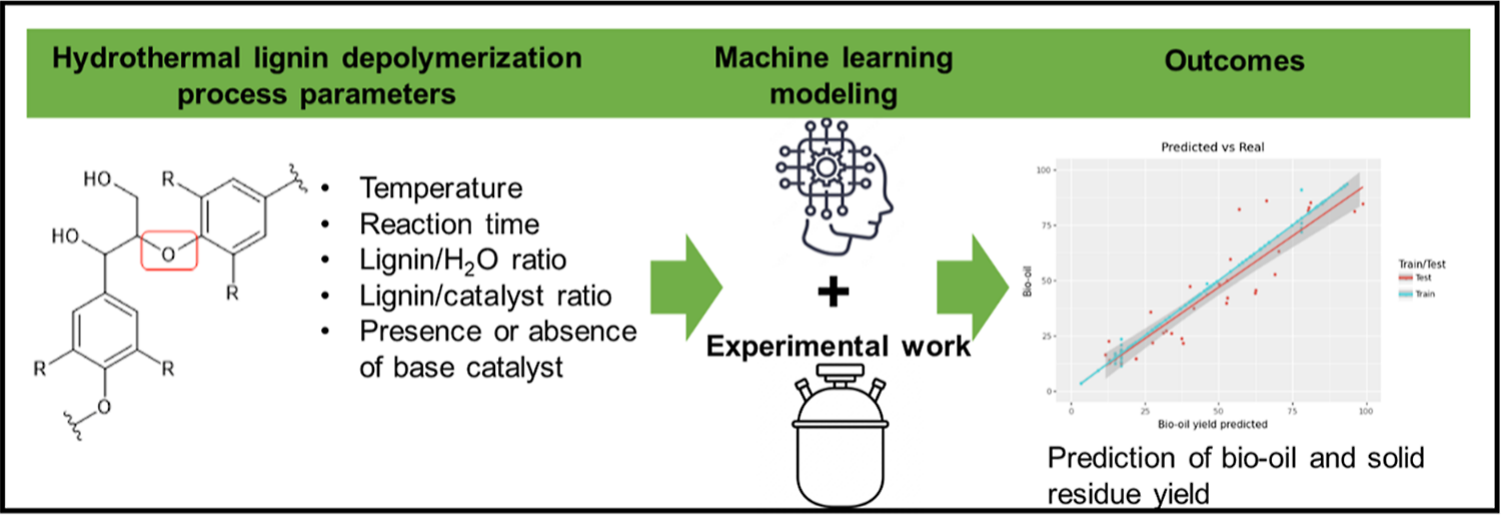

Lignin, an abundant component of plant matter, can be depolymerized into renewable aromatic chemicals and biofuels but remains underutilized. Homogeneously catalyzed depolymerization in water has gained attention due to its economic feasibility and performance but suffers from inconsistently reported yields of bio-oil and solid residues. In this study, machine learning methods were used to develop predictive models for bio-oil and solid residue yields by using a few reaction variables and were subsequently validated by doing experimental work and comparing the predictions to the results. The models achieved a coefficient of determination (*R*^2^) score of 0.83 and 0.76, respectively, for bio-oil yield and solid residue. Variable importance for each model was calculated by two different methodologies and was tied to existing studies to explain the model predictive behavior. Based on the outcome of the study, the creation of concrete guidelines for reporting in lignin depolymerization studies was recommended. Shapley additive explanation value analysis reveals that temperature and reaction time are generally the strongest predictors of experimental outcomes compared to the rest.

## Introduction

Biomass conversion has been suggested as an alternative, CO_2_-neutral source of hydrocarbons and has seen large success over the past decades in the conversion of edible biomass such as sugars to produce ethanol^1^ and oils to produce biodiesel.^2^ However, the conversion of edible biomass has been judged as unsustainable due to its impact on food prices, and, in turn, the usage of non-edible biomass as a feedstock has been suggested instead.^3^ Among these, the conversion of lignocellulose and in particular cellulose has seen the most progress with the emergence of the cellulosic ethanol industry.^4^ Lignin usage, on the other hand, has lagged despite its abundance, accounting for roughly 15 to 25% of the total lignocellulosic biomass available worldwide^5^ and possessing a polymeric aromatic structure that could provide aromatic chemicals currently only available from fossil fuels in any meaningful quantities.^6^ These aromatic chemicals are foreseen to remain a staple of the chemical industry due to their large number of applications in the making of plastics and as precursors for the chemical synthesis of drugs and materials.^7^

Depolymerizing lignin is not an easy task however, as seen from the large body of research in the literature, with numerous studies employing different methods, thermochemical, catalytic, or biological.^8^ Among these, thermochemical and catalytic methods have obtained the largest success, usually obtaining a mixture of gas, solid residue, and bio-oil as products from the reaction, in different proportions depending on the severity of the process, reaction media, and presence or absence of a catalyst.^9^ While the merits of using transition-metal-containing catalysts are well understood, resulting in a higher yield of bio-oil containing less oxygen and lower formation of solid residue,^10^ their application at an industrial scale leaves much to be desired due to their relatively fast poisoning and low cost–benefit ratio when compared to simple strong alkali salts, such as NaOH or KOH in homogeneous reaction media.^11^

Among the existing lignin depolymerization methods, base-catalyzed depolymerization in water as reaction media has attracted attention due to its economic feasibility,^12^ short reaction times,^13^ and reaction performance comparable to that seen in transition-metal-catalyzed processes, in terms of bio-oil yield,^14^ particularly for pulping-derived lignins. Comparatively speaking, base-catalyzed hydrothermal depolymerization is simpler than transition-metal-catalyzed depolymerization in organic solvents due to the lower number of possible interactions among the reactants. This makes base-catalyzed depolymerization seemingly simpler to understand and interpret.

Due to the great number of possible combinations of catalysts, reaction media, and qualities of lignin with different properties, lignin depolymerization research across studies can be perceived as complex or confusing, with many studies reporting very different results that provide only partial or no justification for the choice of metals in the catalyst they use or the reaction media chosen. In previous work,^15^ machine learning (ML) algorithms were used to develop predictive models for yield of bio-oil and solid residue and interestingly, in spite of the large variety of experimental variables and properties, it was revealed that in most cases the predicted yield of bio-oil and solid residue could be attributed to specific process variables, such as temperature, the ratio of lignin to solvent used, and the usage of certain solvent combinations. However, the literature data used in this work represented only a very small fraction of the published data available in the reaction space due to them being gathered only from certain studies that fit the search criteria used in that study. Another recent study^16^ focuses more broadly on predicting and optimizing the results of hydrothermal biomass liquefaction, highlighting the potential in modeling seemingly simple processes to better understand how the process variables impact the experimental results obtained.

Inspired by the recent developments in the usage of ML in the study of thermochemical and hydrothermal conversion of biomass,^17,18^ in this study, base-catalyzed and non-catalyzed hydrothermal depolymerization of lignin was investigated, for the first time, by combining ML modeling to predict the yield of bio-oil and solid residue and experimental work to test the validity of the models. Explainable variable importance for the models was obtained through two different methodologies in order to obtain insight into how the process variables in lignin depolymerization impact the results of the experiments. The results revealed that prediction of solid residues is consistent with the experimental results, but reliably predicting bio-oil yield may be difficult in the absence of clearer and more comprehensive characterization of the lignin used.

## Materials and Methods

### Data Collection and Pre-Processing

A methodology was developed to manually gather data from the existing literature related to hydrothermal and homogeneously catalyzed lignin depolymerization reactions, by utilizing methods published in a previous study.^15^ First, an extensive literature search in Scopus was carried out by using the search string base-catalyzed lignin, finding a total of 146 documents that were then sorted. Of these, 57 documents were downloaded and 12 were selected to obtain the data,^19−30^ resulting in 143 experimental data points captured, out of which 60 were base-catalyzed and 83 were not. In this search, targeted documents contained the variables seen in Table 1; these variables were deemed the minimum necessary reported information required to predict the outcome of a base-catalyzed lignin depolymerization experiment based on existing knowledge in the literature. Of these, reactor volume to solvent volume ratio serves as a rough estimate for the pressure since all studies involved used water as a solvent, and the relative fraction of lignin to water is very small. This study’s leading hypothesis is that the parameters outlined in Table 1 should reliably predict the yield of bio-oil and solid residue from base-catalyzed or not catalyzed hydrothermal lignin depolymerization. Although many studies report the variables outlined in Table 1, many studies contain methodological or experimental variables that deviate from the ones seen in the final data set; notably, the works by Miller, et al.^31,32^ that are considered foundational studies are not included due to them involving the usage of organic solvents. Another notable study by Schutyser, et al.^33^ was considered but ultimately not used as part of the data set due to their usage of high-pressure O_2_. All experiments in the final data set are limited to those involving water reaction media and not involving the use of pressurized air or oxygen.

**Table 1:** ML Features and Label Names, along with Their Descriptions

feature and label names	description
lignin/H_2_O ratio	ratio of lignin to solvent in the experiment (mg/mL)
lignin/catalyst ratio	ratio of lignin to the homogeneous catalyst used in the experiment (wt/wt)
catalyzed or uncatalyzed^a^	whether the experiment was catalyzed or not
reactor volume/H_2_O ratio	ratio of the volume of the reactor used to the volume of solvent in the experiment (vol/vol)
reaction time	reaction time in seconds
temperature	temperature in K
bio-oil yield^b^	bio-oil yield from lignin (wt %)
solid residue yield^b^	solid residue yield from lignin (wt %)

a This variable was one-hot-encoded due to its categorical nature.

b Bio-oil and solid residue yield are labels.

“Solid residues” was chosen instead of “char” due to the ambiguity related to the terminology used; in many studies, they use the words char, coke, and repolymerized lignin without clear consistency; thus, “solid residues” represents all of the solids obtained post-reaction. All data were manually extracted from the chosen studies either directly from tables or graphs by using graph digitizing software.

Originally, gas yield was also intended to be captured; however, gas yield was inconsistently reported in papers and thus deemed not fit to be used in this study.

**Figure 1:**
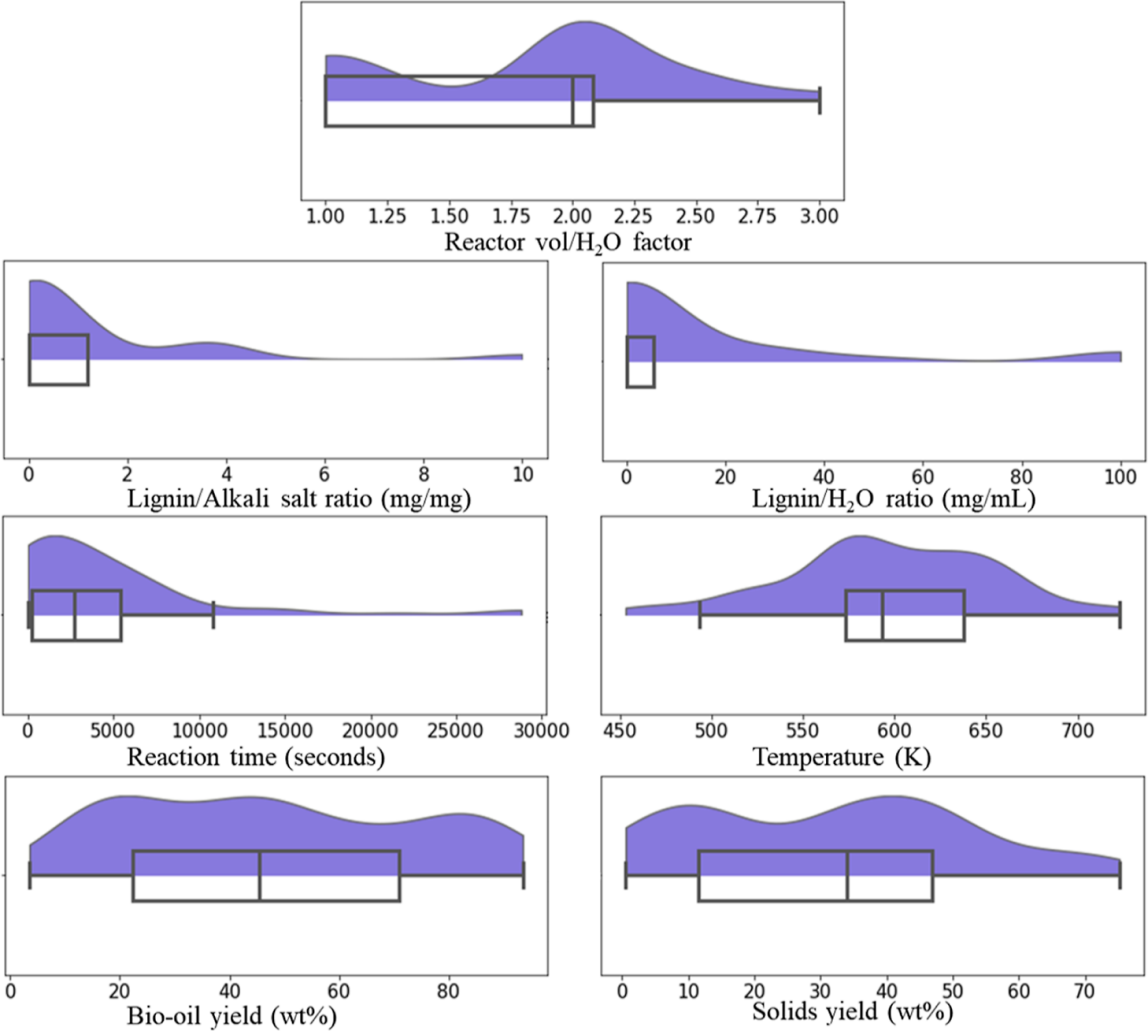
Compiled violin plots for features and labels captured from the literature.

In order to further clarify the distribution of the data captured from literature, violin plots were elaborated for all features and labels, illustrated in Figure 1.

**Figure 2:**
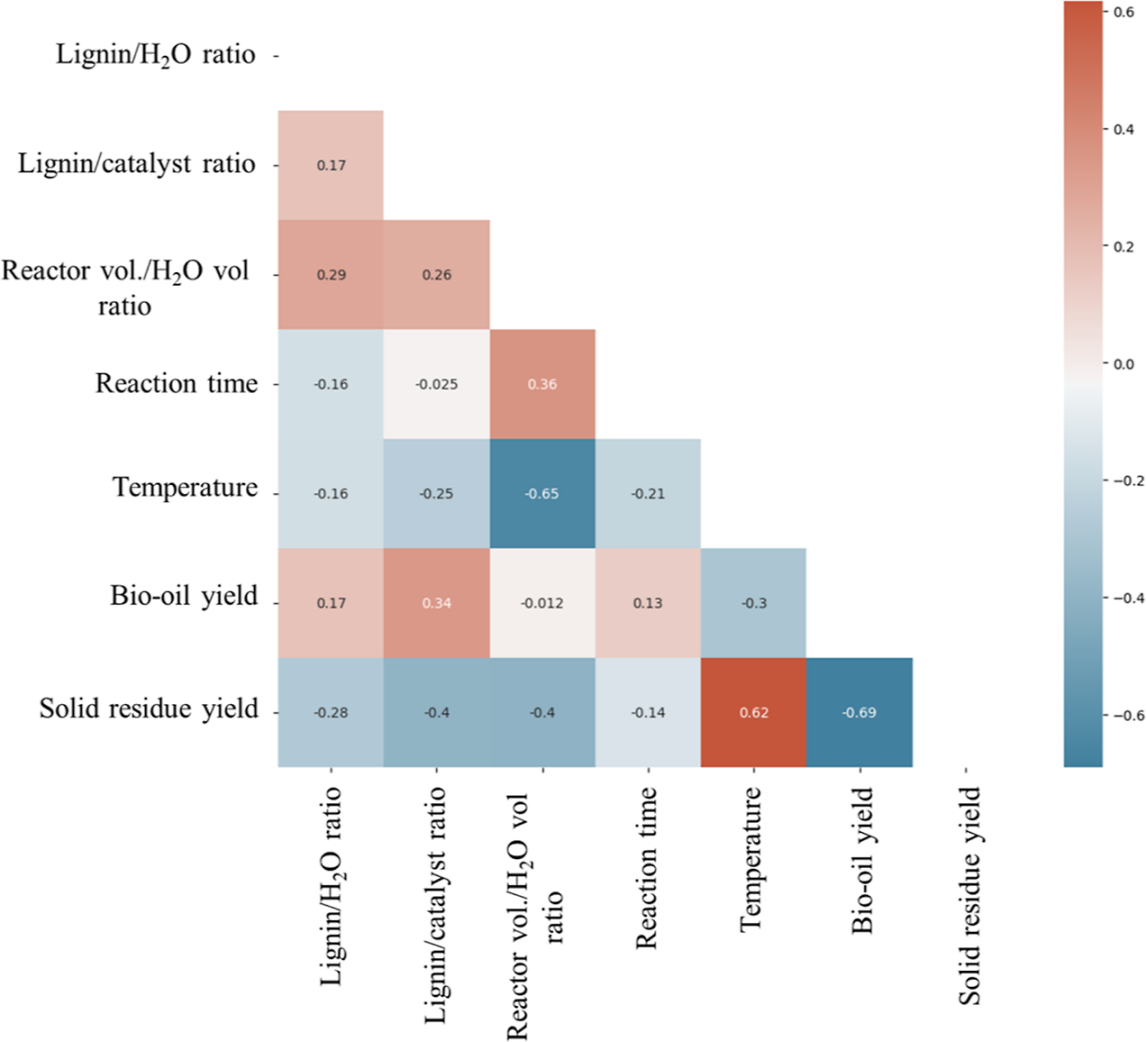
Spearman’s rank correlation heatmap for data captured from the literature.

It is clear from Figure 1 that the distribution of the data capture is most often not normal, and in the case of reaction time, lignin/catalyst ratio, and lignin/H_2_O ratio, they were heavily skewed toward low values. While it is worth noting that most ML methods do not rely on assumptions of normal distribution in the data used to work properly, it does demonstrate that the experimental parameters found in the literature are less diverse than one could imagine. This is most likely due to the natural tendency of researchers to further test experimental conditions that yielded good results for others, though it must be stated that it is not representative of the entire possible experimental space. Due to the lack of normality in the data captured, Spearman’s rank correlation was used to analyze the data and is presented in Figure 2.

While a clear correlation can be observed between the yield of bio-oil and solid residue due to their mutually exclusive nature, most other correlations are weak. Temperature, however, shows a very strong positive correlation with solid residue yield, which makes sense based on previous trends in the literature.

During the data gathering phase, two assumptions were made: first that the catalytic activity of different alkali salts is roughly the same, with NaOH, KOH, and Na_2_CO_3_ being featured in this data set. It is foreseen that this simplification would be a source of inaccuracy in the model, as it is known that there are measurable differences amongst different alkali salts with different lignins^34^ The second assumption is that heating rate did not play a significant role in the outcome of the experiment. It was chosen to not extract these data as they were often not reported in the selected studies or could not be inferred reliably in the case of continuous flow experiments.

### ML Methods, Evaluation Indicators, and Feature Importance Calculation

**Figure 3:**
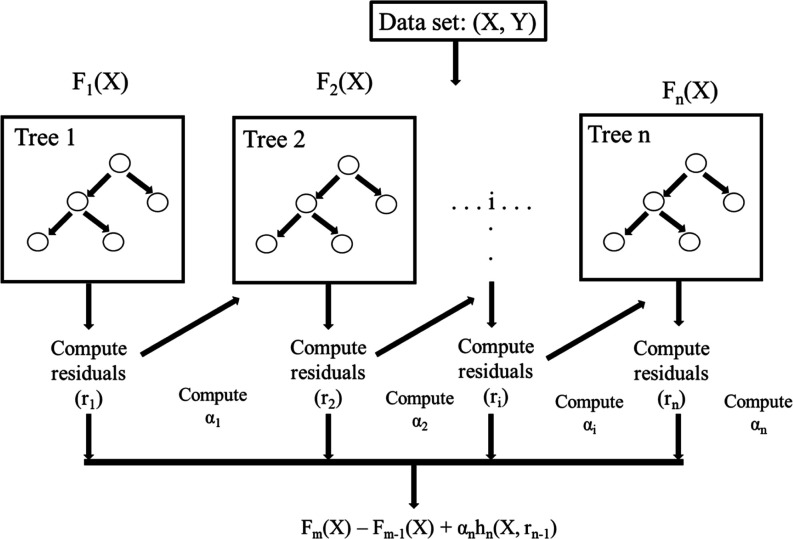
Representation of how an XGBoost model works.

Scikit-learn free ML libraries for python were used to implement Extreme gradient boost regression (XGBoost).^35^ Other decision tree-based models (Random Forest, Gradient Boosting Regression, AdaBoost) were tested but XGBoost showed a marginally higher performance. XGBoost is an ensemble algorithm that employs decision trees as weak learners and was chosen due to its ease of understanding, lack of necessity for data to follow a normal distribution, and capacity to handle continuous and categorical data. Most importantly, the algorithm was robust against overfitting, outliers, and noise.^36^ In Figure 3, a diagram of how an XGBoost model works is shown.

α_*i*_ and *r*_*i*_ are the regularization parameters and residuals computed with the *i*th tree, respectively, and *h*_*i*_ is a function that is trained to predict the residuals *r*_*i*_ using *X* for the *i*th tree. To compute α_*i*_, the residual-computed *r*_*i*_ are used and the following is computed, as shown in 1
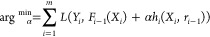
1where *L*(*Y*, *F*(*X*)) is a differentiable loss function.

The reason for only decision tree-based models being tested is the heterogeneous nature of the data collected from multiple studies that may include implicit or explicit differences in terms of experimental practices. Purity of reactants and lignin used as well as differences in reactor design and workup were accounted for as much as possible in the data gathered; however, there may be differences or characteristics not explicitly stated in the studies where the data come from. Additionally, XGBoost has the benefit of acknowledging sparse data distributions when assigning scores, which is relevant due to the nature of the data used in this study, as seen in Figure 1, where some of the distributions are heavily skewed in one direction due to the large number of instances of “0”.

The data available were separated into training and testing data, accounting for 75 and 25% of the total, respectively; 5-fold cross-validation was carried out in each instance to observe the bias and variance in each case. The performance of the models was evaluated by using coefficient of determination (*R*^2^) and root-mean-squared error (RMSE), whose equations are shown below, 2 and 3.
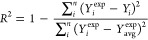
2
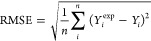
3where *n* represents the number of test samples, *Y*_*i*_^exp^ denotes the experimental value, and *Y*_*i*_ represents the predicted value. *Y*_avg_^exp^ represents the mean value of *Y*_*i*_^exp^ and *Y*_*i*_, respectively.

Feature importance for the models was obtained both by mean decrease in impurity (MDI) and SHAP (Shapley Additive explanations) values. MDI calculates the total reduction in loss or impurity contributed by all splits for a given feature. This method is computationally very efficient and has been widely used in a variety of applications.^37^ SHAP values include a method of feature importance estimation based on cooperative game theory where each feature is interpreted as a “player” and is used to increase transparency and interpretability of ML models.^38^ Partial dependency plots were used estimate the impact of using different temperatures and reaction times on the yields of bio-oil and solid residues.

## Materials

Dealkaline lignin (DL) was bought from Asahi Kasei Chemicals, Japan. DL was completely soluble in aqueous alkali solutions of pH > 10. Other chemicals include NaOH (98%), acetone (98%), dichloromethane (99.8%) (DCM), and hydrochloric acid, all bought from Wako Chemicals, Japan.

### Base-Catalyzed Lignin Depolymerization Experiments

Depolymerization experiments were carried out in 50 mL Taiatsu (Japan) TPR-5 reactors equipped with a pressure gauge, thermocouple, and gas line for experiments at 250 °C; for experiments at 350 °C, self-made autoclave reactors were used. Across all experiments, 125–250 mg of DL was loaded in the reactors, and then a varying amount (10 to 30 g) of water and 125 to 250 mg of NaOH were added. The reactor was then sealed and heated using heating jackets at an average of 10 °C/min until reaching the target temperature. Reaction time includes the heating ramp. During the course of the experiment, pressure increased from atmospheric pressure to 3–5 MPa mainly due to water vapor pressure, as the amount of gas produced by the lignin was comparatively smaller. After the reaction time had elapsed, the reactors were quickly cooled down by using an electric fan until the temperature dropped below 50 °C. The reactors were then opened to release the gas products and the liquid and solid products were then dumped into a beaker. The reactors were thoroughly washed and scrubbed with distilled water to remove any particles from the walls of the reactors.

The liquid and solid products were then acidified by using 2 M HCl until the pH reached 1–2 to precipitate lignin oligomers. Subsequently, the products were filtered with a pre-weighted 110 mm filter paper, and the aqueous fraction was combined with 30 mL of DCM and thoroughly mixed to extract any present water-soluble aromatics. The aromatics in the DCM solution were later separated by using a rotary evaporator. Precipitated lignin was washed with acetone to separate acetone-soluble products (ASP), which are reported as part of the bio-oil yield. Yields of bio-oil and solid residue were defined as follows
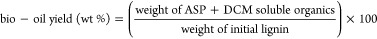
4
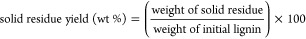
5

The gas fraction produced was not captured nor analyzed in these experiments.

## Results and Discussion

### Evaluation of ML Model Performance for Bio-Oil Yield Prediction

**Figure 4:**
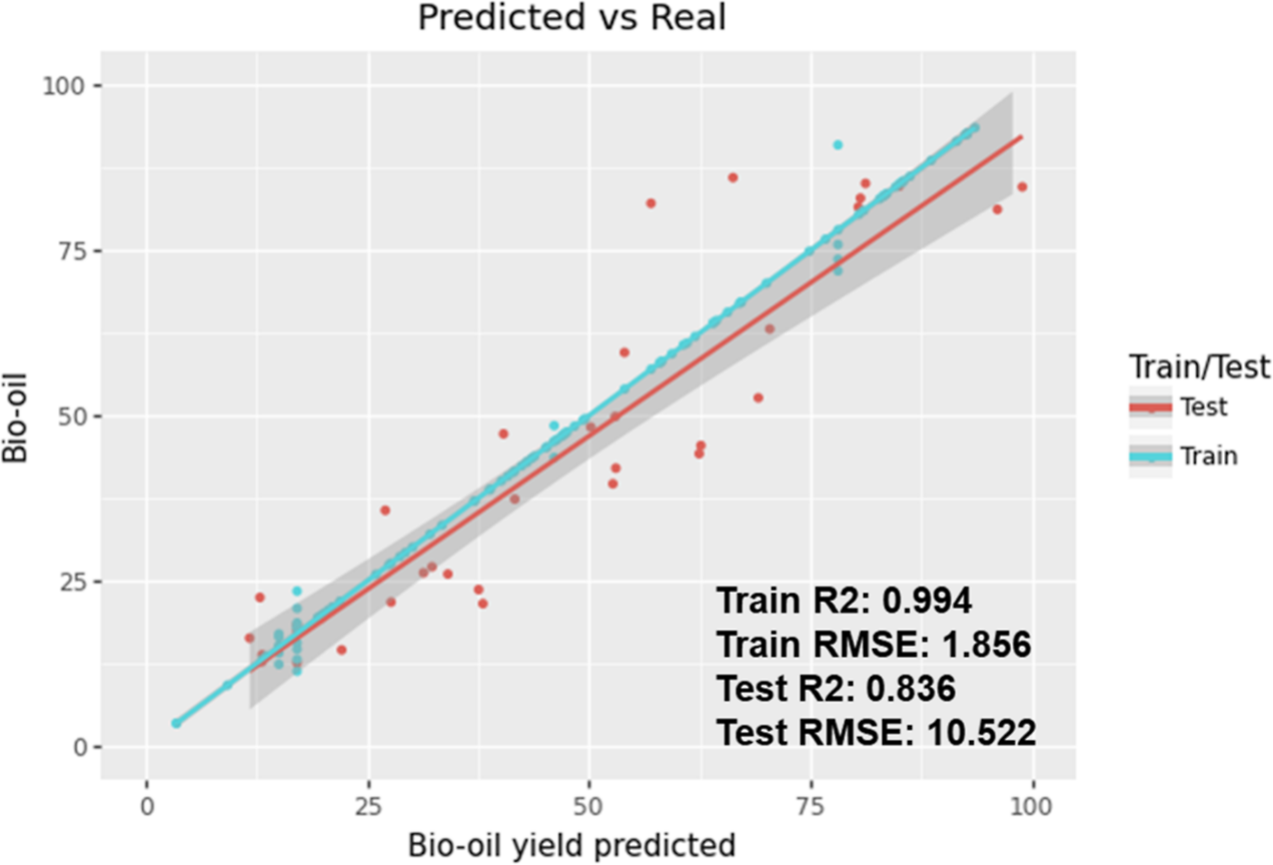
Prediction performance of the XGBoost model for bio-oil yield and its associated RMSE and *R*^2^ scores.

Using the literature data, a prediction model for bio-oil yield was developed by first removing the yield of solid residues from the data sets, as bio-oil, solid residue and gas share a zero-sum relationship as lignin is the only reactant in these experiments. Shown in Figure 4, the prediction performance scores for the model developed can be observed with an RMSE of 10.522 and *R*^2^ score of 0.836. The gray band indicates the 95% confidence interval.

The model displayed better prediction capability at low and middle bio-oil yields (20–60 wt %), with predictions on the higher end accounting for most of the RMSE.

Based on this, the MDI variable importance is also shown in Table 2, where reactor volume to H_2_O volume ratio holds the highest importance followed by lignin to H_2_O ratio, lignin to catalyst ratio, temperature, and reaction time, in that order.

**Table 2:** MDI Feature Importance for Prediction on Bio-Oil from the XGBoost Model

feature	importance
reactor vol/H_2_O vol ratio	0.600
lignin/H_2_O ratio (mg/mL)	0.189
lignin/Catalyst ratio	0.079
temperature	0.079
reaction time	0.050

These variables are known to have an impact on the outcome of the experiment based on the existing knowledge in the literature; however, the magnitude at which they do is not clear when comparing one variable against another. Though the MDI importance values shown reflect “how important” one variable is compared to other, it must be kept in mind that this variable importance only pertains to a limited part of the possible experimental space.

It is well understood that temperatures close to the critical temperature (647.14 K) of water can offer higher yields of bio-oil.^24^ Simultaneously, when the temperature exceeds the critical temperature of water, a higher likelihood of gas-forming reactions taking place is also a possibility, in both the presence and absence of catalysts.^39^

Regarding the importance of the ratio of lignin to solvent, it stands to reason that re-polymerization behavior that results in the formation of solid residues is intensified when the concentration of lignin is high, and because of the zero-sum relationship between bio-oil, solid residue, and gas, it makes sense that it shows high importance; it is also a behavior seen in lignin solvolysis studies.^40^

The ratio of lignin to catalyst, on the other hand, is a more complicated issue, with various reports on what is the optimal concentration of catalyst in their experiments^41^ from 2 to 4 wt % based on the quantity of lignin; these differences could be attributed to the nature of the lignin used in the experiments, as it is known that depending on the method used to isolate the lignin, its solubility in water or other solvents varies,^42^ thus impacting the results.

The importance of reaction time is difficult to assess, as the published literature data used in this study included experiments with reaction times as short as 0.5 s;^23^ however, due to the homogeneous nature of these experiments, it is reasonable to assume that there are no mass transfer limitations with the catalyst, and the severing of C–O–C bonds is known to be a fast reaction when using model compounds;^43^ it is likely that at least partial depolymerization into bio-oil-like mixture of monomers and oligomers can be achieved even with short reaction times.

The ratio of reactor volume to H_2_O volume holds most of the importance. This ratio correlates to the operational pressure at a given temperature; however, because of this, its impact is also dependent on the temperature of the experiment.

**Figure 5:**
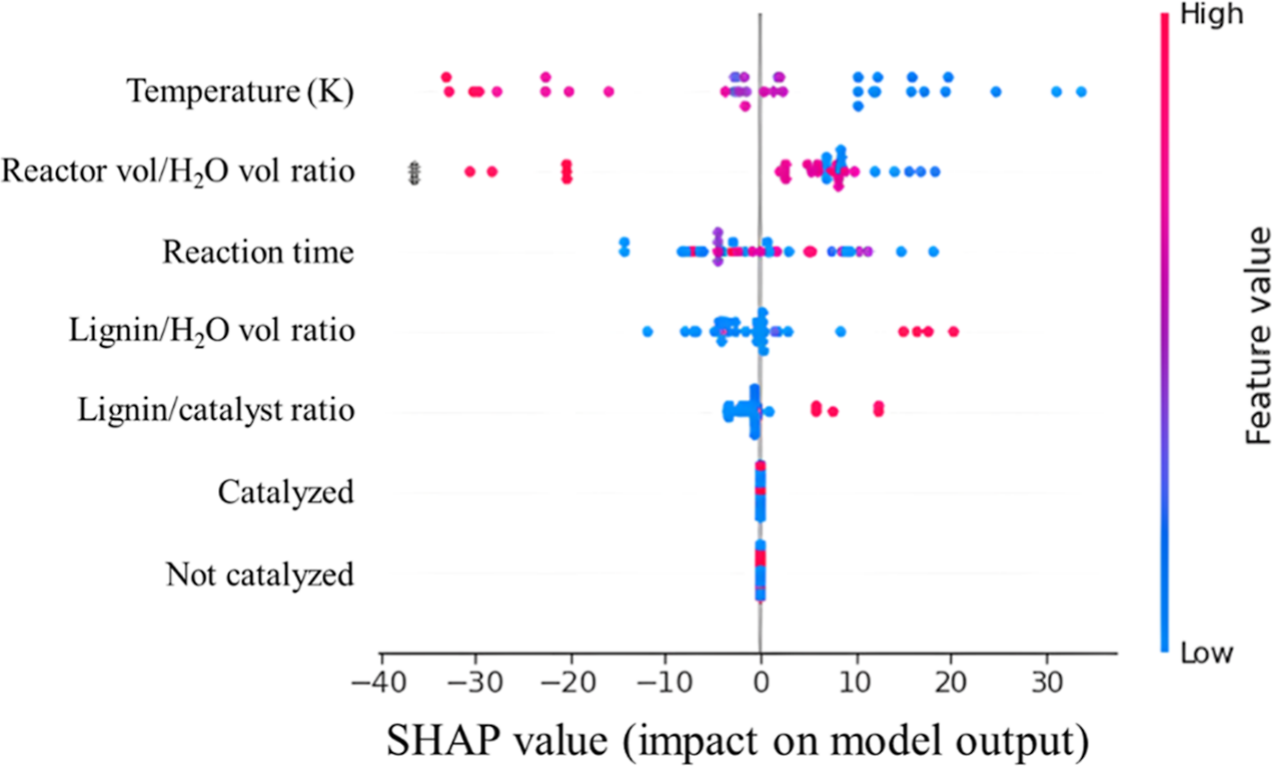
SHAP values beeswarm plot for the XGBoost bio-oil prediction model.

In contrast to Table 2, in Figure 5 a beeswarm representation of the SHAP values obtained for the same model that also represent the importance of the features in the model are shown. In this plot, features are ordered from top to bottom in order of magnitude of importance in the vertical axis; in the horizontal axis, the magnitude of positive or negative contribution toward the predicted bio-oil yield can be seen, mediated by the color of the points, where red means high and blue means low. It is immediately clear that the order of the features in Figure 5 is not the same as that shown in Table 2 due to the way SHAP and MDI are calculated.

The beeswarm plot in Figure 5 allows for interesting observations regarding temperature, as it indicates that very high values negatively impact the yield of bio-oil, which in the case of this study would mean 663–723 K, approaching gasification temperatures, while purple to blue values can be positively correlated with high bio-oil yield. Similarly, in terms of the ratio of lignin to alkali salt, a low to middle value appears to have either no impact or negative impact on the bio-oil yield, while a high loading of alkali salt increases bio-oil yield, presumably by increasing lignin solubility and guaranteeing interaction with the catalytically active OH^–^ ion the reaction media. The interpretation of the reactor volume to water volume ratio indicates that a low volume of water within the reactor with regard to the total volume available is negatively correlated with bio-oil yield. This ratio is meant to serve as a proxy for pressure inside the reactor under the assumption that most of the pressure during the process can be attributed to the presence of water at high temperatures and not necessarily due to gas products. The interpretation of lignin to solvent ratio and reaction time is complicated as both high and low values can be seen over the horizontal axis. However, it is important to note that this means there are interactions between variables that allow for this to be true, particularly for the absence/presence of a catalyst, for example, that a short reaction time in the presence of a high alkali salt concentration could still result in high bio-oil yield or that repolymerization into solid residues is minimized in the presence of enough alkali salts, thus leading to higher bio-oil yield as a co-consequence.

**Figure 6:**
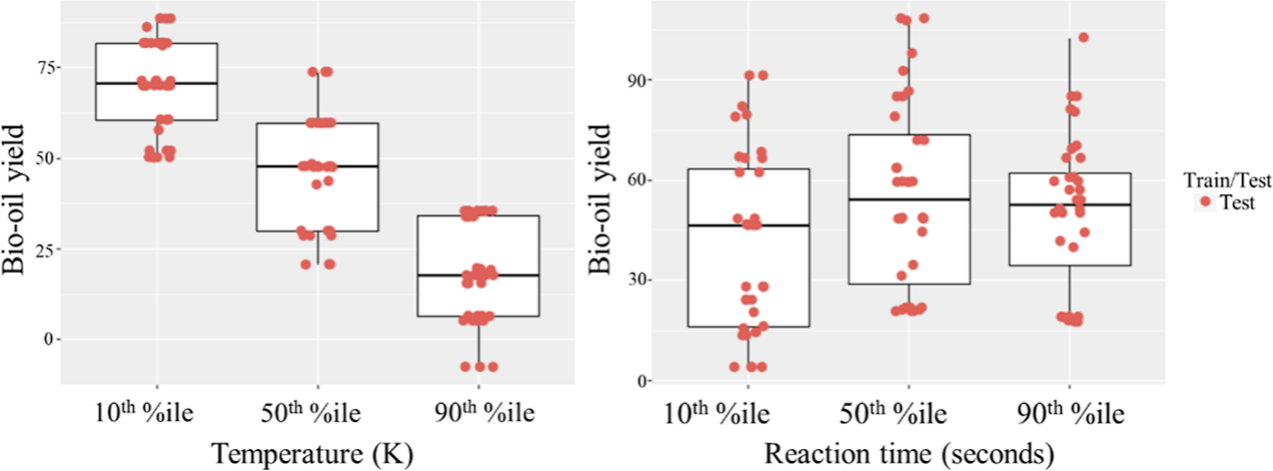
Partial dependency plots for temperature and reaction time impact on bio-oil yield.

Of the controllable variables in a given lignin depolymerization experiment, process temperature and reaction time are thought of as the most critical and straightforward directly controllable process parameters. In Figure 6, partial dependency plots that illustrate the impact of temperature and reaction time on bio-oil yield are shown, where boxplots show the possible range of bio-oil yield for the various experiments in the data set under the assumption that only temperature is changed to the values seen in the 10th, 50th, and 90th percentiles of process temperature found in the data set. For temperature, a clear tendency can be observed for predicted bio-oil yield to decrease as temperature increases, which makes sense given that the higher temperature values in the data set approach gasification temperatures, which results in the production of non-condensable gases at the expense of bio-oil yield and solid residue yield. For reaction time, a clear trend cannot be observed, with predicted bio-oil yield values fluctuating between slightly lower and then higher as the reaction time moves from the 50th percentile to the 90th. It is possible that the strongly skewed, non-normal distribution of reaction time in the data set may negatively affect the interpretability of this variable.

### Evaluation of ML Model Performance for Solid Residue Yield Prediction

**Figure 7:**
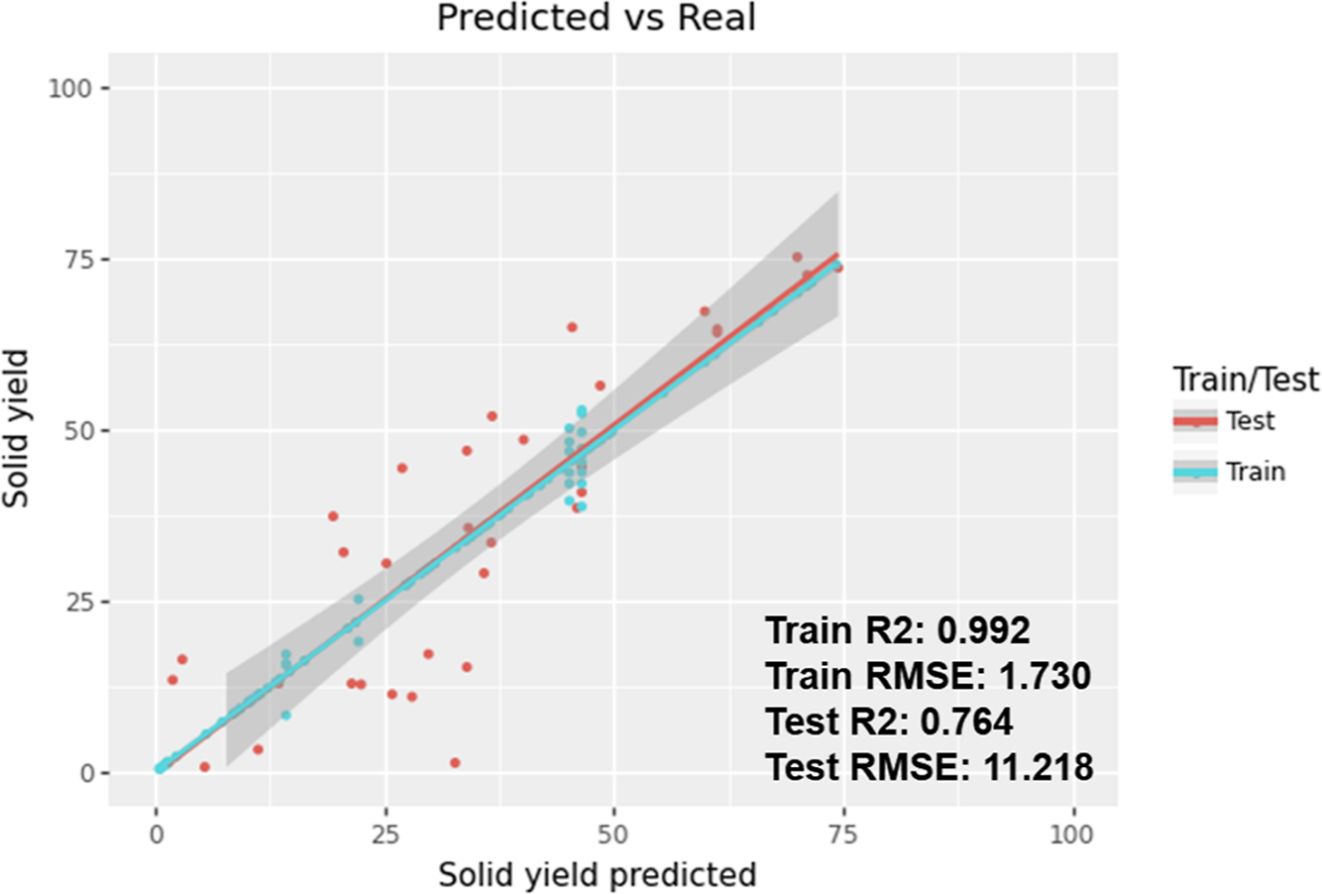
Prediction performance of the XGBoost model for solid residue yield and its associated RMSE and *R*^2^ scores.

The ML prediction models for solid residue were developed by first removing the yield of bio-oil from the data sets, as bio-oil, solid residue, and gas share a zero-sum relationship. In Figure 7, where the prediction performance of the model is displayed as an *R*^2^ score and RMSE value, the XGBoost model can be seen with an *R*^2^ score of 0.764 and an RMSE of 11.218. The gray band in the graph represents the 95% confidence interval.

The MDI variable importance for the prediction of solid residues is shown in Table 3, where the magnitude and order of the features seen are different from that observed for bio-oil prediction. While it is not possible to extrapolate from these values which temperature is optimal for minimizing the yield of solid residue, within the context of the study, there are data from experiments where low temperatures resulted in a higher likelihood of re-polymerization of lignin.^24^ Note that in the data gathering, repolymerized lignin, char, and coke were not distinguished, as different authors had used these terms disregarding their definition.

**Table 3:** MDI Feature Importance for Prediction on Solid Residue Yield from the XGBoost Model

feature	importance
lignin/H_2_O ratio (mg/mL)	0.740
reactor vol/H_2_O vol ratio	0.150
temperature	0.050
reaction time	0.039
lignin/Catalyst	0.029

Additionally, it is also possible that traces of cellulose may contribute to the formation of char,^44^ and various studies included in the data set use lignins that may contain such traces.^19,27^ In a similar manner to bio-oil yield, a high concentration of lignin in the reaction media may result in a higher likelihood that the species responsible for the formation of repolymerized lignin and char react to form a more solid residue. It is known in the literature that to allow the maximum possible lignin concentration in pilot-scale processes, formaldehyde-reacting chemical species are co-fed to the reactor, such as phenol.^41^

**Figure 8:**
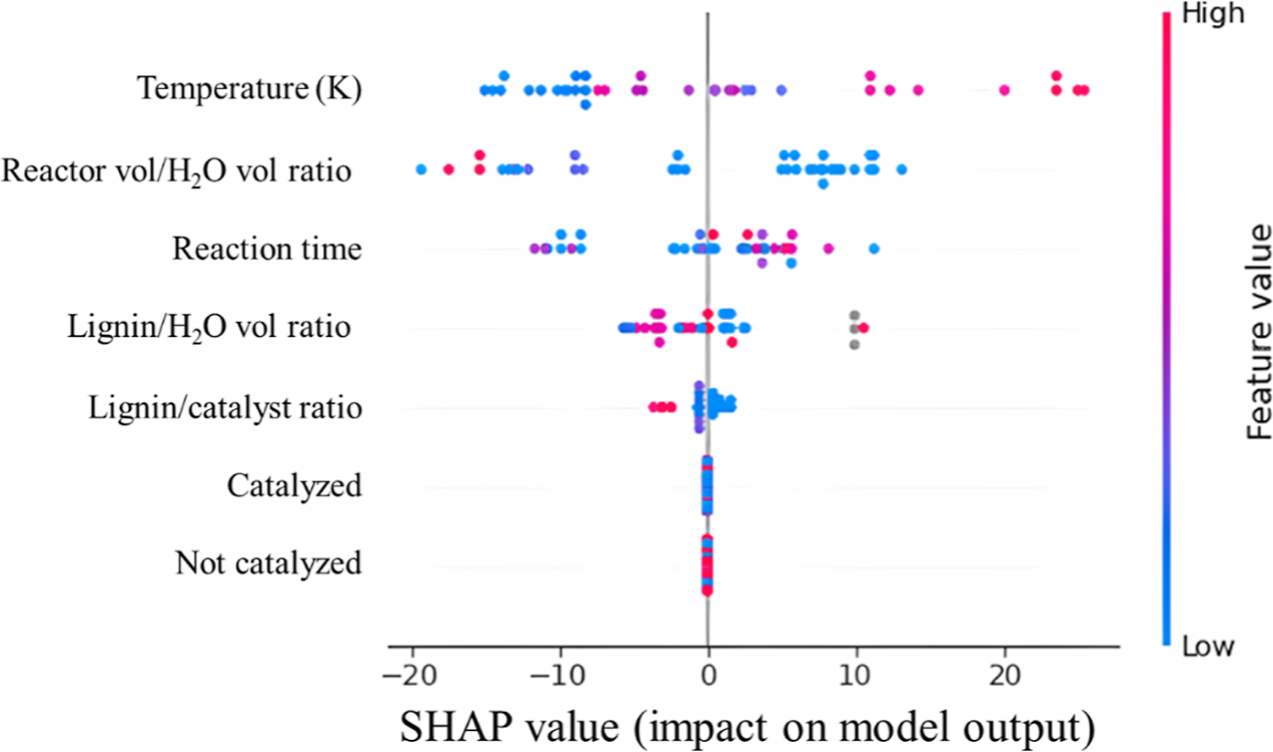
SHAP values’ beeswarm plot for the XGBoost solid residue prediction model.

In contrast to the values shown in Table 3, the beeswarm plot for SHAP values shown in Figure 8 offers a much more detailed and nuanced perspective on feature importance than that obtained from MDI in Table 3. Here, it can be observed that much like in the case of bio-oil yield, especially high temperatures are associated with higher yields of solid residue, being consistent with the zero-sum nature of bio-oil, solid residue, and gas yield. High reaction times tend to be associated with higher solid residue yields, though not always, as there are some instances of high reaction times that did not particularly affect the yield of solid residue in either direction, again, indicating in those cases that an interaction between variables happens, which allows for this to be possible. It is worth noting that the ratio of reactor volume to water volume in the experiment does not seem to play a large role in solid residue prediction, like it did for bio-oil yield, and that both lignin to catalyst and lignin to solvent ratios show a pattern opposite of that seen in the beeswarm plot for bio-oil yield prediction in Figure 5.

**Figure 9:**
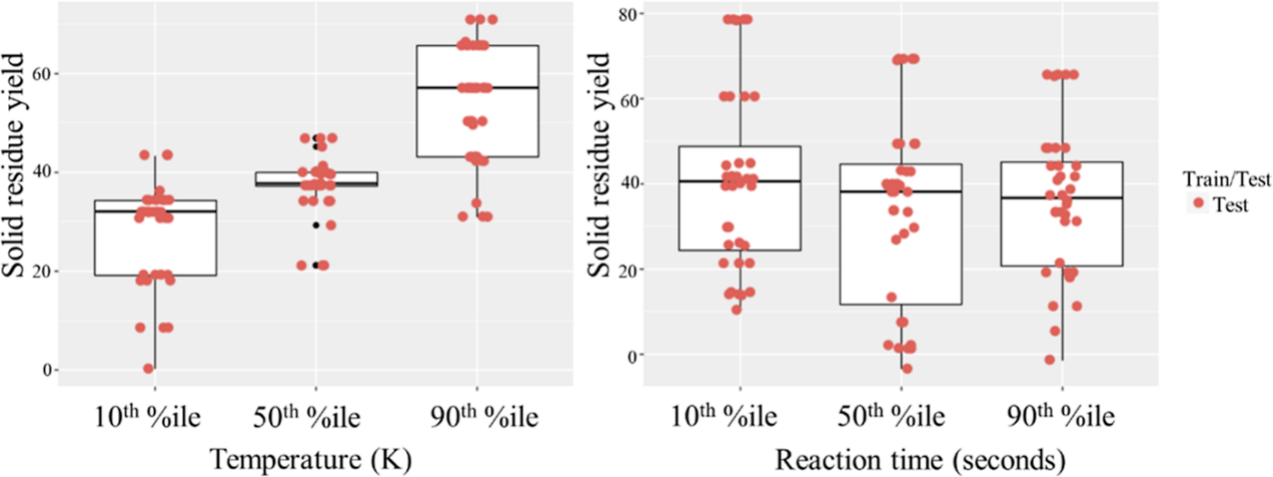
Partial dependency plots for temperature and reaction time’s impact on solid residue yield.

In Figure 9, a partial dependency plot displaying the effect of process temperature and reaction time on solid residue yield is shown. Higher temperature seems to be clearly associated with higher yield of solid residues; however, at the temperature found in the 50th percentile, a very erratic boxplot can be seen, which includes a few outliers (black dots). It is unclear why the predicted values are like this. Given that the temperature distribution is relatively normal-shaped and data are most abundant around the 50th percentile, it is most likely not due to data sparsity. Reaction time, on the other hand, shows a decreasing trend as reaction time increases. As previously mentioned, this may be due to lignin’s tendency to repolymerize as reaction time increases in particular circumstances. There may also be an interaction effect between temperature and reaction time with regard to solid residue yield, where higher temperatures result in higher solid residue yield if reaction time is extended.

### Experimental Validation of Predictive Models

In order to further validate the predictive models made and obtain insight into the experiments they are based on, a series of experiments were carried out as shown in Table 4, with the experimental results reported being the average of triplicate runs. All experiments were carried out with NaOH as the catalyst due to it being the most commonly seen in the published literature. Temperatures of 250 and 350 °C were chosen for the experiments due to them being the most representative within the data set and also because of limitations on the temperature and pressure limits of the reactors used. As seen in Table 4, the experimental results can deviate strongly from the predictions made with the models, particularly in regard to bio-oil yield, with prediction of solid residues falling within the expected prediction error range of the trained model.

**Table 4:** Experimental Validation of Predictive Models for Bio-Oil Yield and Solid Residue Yield

T (°C)	reaction time (h)	lignin–solvent ratio	lignin–catalyst ratio	reactor volume–solvent volume ratio	experimental bio-oil yield (wt %) (E. bio-oil yield)	predicted bio-oil yield (wt %)	prediction gap for bio-oil^a^	experimental solid residue yield (wt %)	predicted solid residue yield (wt %)	prediction gap for solid residue^a^
250	1	16.66	1	3.33	28.9	57.93	29.03	45.38	39.57	5.81
250	3	16.66	1	3.33	19.70	71.43	51.73	41.25	43.51	2.26
250	1	16.66	0.5	3.33	35.15	55.68	20.53	30.75	39.37	8.62
250	3	16.66	0.5	3.33	24.74	69.07	44.33	37.01	43.54	6.53
250	1	16.66	0.5	1.66	24.20	91.17	66.97	39.75	20.46	19.29
250	3	16.66	0.5	1.66	20.08	84.9	64.82	37.34	25.18	12.16
350	1	12.5	1	2	24.8	58.98	34.18	44.8	27.90	16.9
350	3	12.5	1	2	19.6	61.38	41.78	24.8	27.28	2.48
350	1	25	2	2	16.53	58.92	42.39	30.8	29.40	1.4
350	3	25	2	2	14.8	55.37	40.57	18.66	33.83	15.17
350	1	25	1	2	32.8	58.92	26.12	24.13	29.40	5.27
350	3	25	1	2	15.53	54.46	38.93	23.00	33.83	10.83

a “Prediction gap” is defined as the absolute difference between the model prediction and the experimental result.

Considering the results obtained from the experimental work, literature was consulted to find experimental reasons as to why the bio-oil yield could vary across studies despite using the same or very similar reaction conditions. Two main sources of variation can be pointed at, first, the difference of solubility of different lignins in NaOH solutions^42^ partly due to different phenol hydroxyl group contents which impact the way the reaction occurs. Second, upon comparison of the various methodologies involved in the studies used as a source of data for the modeling, it is clear that the choices made in the post-reaction handling of the lignin products play a large role in the resulting calculated yield of bio-oil and solid residue. The recovery of the soluble lignin phase by using an organic solvent and whether that is considered part of the bio-oil yield or not are questionable. From the data science-perspective, the data set used contains multiple experimental instances where the results fluctuated heavily for bio-oil yield at a given temperature and time, which may hinder the predictions made by the model.

### Recommendations Based on the Current and Previous Works

This study is the first attempt to use ML to predict experimental outcomes in base-catalyzed or uncatalyzed hydrothermal lignin depolymerization.

The results reveal that significant predictive performance can be achieved for solid residues from the experiments, but bio-oil yield shows a large prediction gap when compared to the experiments done to test the model.

Previous work on predicting lignin depolymerization performance in heterogeneously catalyzed reactions^15^ included more reaction parameters, yet achieved higher prediction performance. In Table 5, a comparative table is shown, which includes previous work in predicting bio-oil yield in heterogeneously catalyzed lignin depolymerization and hydrothermal liquefaction of various feedstocks.

**Table 5:** Comparison of Model Bio-Oil Yield Predicting Performance with the Previous and Related Recent Literature

feedstock and conversion method	model used	data used	input features	test *R*^2^	test RMSE	refs
lignin; base-catalyzed/non-catalyzed hydrothermal liquefaction	extreme gradient boosting	143	6 features; temperature, residence time, lignin/H_2_O ratio, lignin/catalyst ratio, reactor vol/H_2_O ratio, based-catalyzed (catalyzed/uncatalyzed)	0.83	10.52	this study
lignin; heterogeneously catalyzed liquefaction	random forest	102	6 features; temperature, residence time, solvent choice, active metal/lignin ratio, catalyst/solvent ratio, and active metal/solvent ratio	0.90	6.03	(15)
lignocellulosic waste; hydrothermal liquefaction	random forest	117	10 features; elemental composition (C, H, N, O, and S), atomic ratio (H/C, O/C, and N/C), temperature, and residence time	0.85	5.83	(45)
algae; hydrothermal liquefaction	gradient boost regression	310	15 features; elemental composition (C, H, N, O, and S), atomic ratio (H/C, O/C, and N/C), biological composition (content of protein, lipids, or carbohydrate), temperature, and residence time and ash content	0.90	4.69	(46)
wet biomass and wastes; hydrothermal liquefaction	extreme gradient boosting	325	17 features; elemental composition (C, H, O, and N), atomic ratio (H/C, O/C, and N/C), biological composition (content of protein, lipid, or carbohydrate), ash content, residence time, temperature, initial pressure, reactor size, biomass loading, and water and water to biomass ratio	0.87	5.41	(47)
various types of biomass waste; hydrothermal liquefaction	Gaussian process regression	652	10 features; elemental composition (C, H, O, N, and S), ash content, operating dry matter, temperature, residence time, and pressure	0.95	0.038	(16)

From Table 5, it can be seen that the prediction performance for bio-oil in this study is lower than those seen in other recent studies. It must be kept in mind that the other studies save for^15^ focus on hydrothermal liquefaction of non-lignin feedstocks and do not involve the use of catalysts. However, what can be appreciated across these other studies is that comprehensive characterization of the feedstock used is associated with better prediction performance, for the most part. Many lignin depolymerization studies (including the ones used for data gathering in this study) do not characterize the lignin used, which could partly explain the gap in performance. From the perspective of SHAP values, this study’s practical interpretability is high due to its focus on purely operation conditions, instead of intrinsic properties of the feedstock such as that seen in^45^ where a marginally higher *R*^2^ score was obtained but relies largely on feedstock’s properties to predict the yield of bio-oil.

This study and the prior studies both fail to account for the fact that while bio-oil yield may be predictable, the more important target of lignin depolymerization is the yield of aromatic monomers obtained from lignin. Predicting aromatic monomer yield is a more difficult task, as very few publications do comprehensive quantification of the monomers obtained and often use different methodologies to do so, for example, only analyzing the monomers in the aqueous phase of the experiment, the light oil phase, or all the phases, sometimes not explicitly stating what they did too. Regardless, the possibility of predicting the yield of aromatic monomers from a given lignin depolymerization experiment will depend not only on the reaction parameters used but also on the inherent chemical structure of the lignin used, as the upper limit of aromatic monomers obtainable depends on the magnitude of C–O–C bonds present in the lignin, as C–C bonds are often impossible to break during most thermochemical processes that do not involve a noble metal catalyst^48^ or use temperatures that border on gasification. These chemical bonds can be quantified by nuclear magnetic resonance.^49^

Modeling the impact of homogeneous base catalysts such as the ones involved in this study represents a different challenge from that seen in other studies that attempt to describe material properties through the use of various intrinsic-measurable properties such as surface area and pore properties or those based on chemistry principles such as adsorption energies on transition-metal surfaces.^50^ In contrast to this, the description of catalytically active chemical bases (NaOH for example) could be simpler as the role that the Na^+^ and the OH^–^ ions play is well understood^51^ and would require fewer descriptors.

Additional data are always desired when creating these kinds of predictive models. However, since each data point comes from a single experiment, it is understandably difficult to gather data in the magnitudes seen in other areas where ML modeling has been applied, such as sales, weather, or classification of pictures. An emergent approach that could resolve this issue is using at least partially simulated data from experiments as part of the data used. However, complete simulation of lignin depolymerization processes has not been found in the literature, with the exception of studies that focus purely on theorical interactions of specific chemical bonds with catalysts^52^ but not the complete depolymerization process itself perhaps due to the complexity of simulating heterogeneous polymers interacting with a solid catalyst.

Although this study does not deal with the lignin-first biorefinery concept, a recent work by^53^ outlines extensive guidelines for the analyzing of data from lignin-first approaches, including feedstock analysis and process parameters, with the ambition of uniting the lignin-first research community around a common set of reportable metrics. These guidelines comprise standards and best practices or minimum requirements for feedstock analysis, stressing reporting of the fractionation efficiency, product yields, solvent mass balances, catalyst efficiency, and the requirements for additional reagents such as reducing, oxidizing, or capping agents.

These guidelines and minimum reporting requirements when publishing a paper can potentially allow for easier usage of data from literature in future ML-lignin depolymerization-related studies, by guaranteeing that data across studies are compatible with each other. The formation of the guidelines needs further discussion in academic societies or perhaps in this journal in order to advance the lignin depolymerization ML field. Whether the guidelines described in^53^ would be partially or entirely compatible with homogeneously catalyzed lignin depolymerization studies is an issue that needs to be analyzed critically.

## Conclusions and Future Directions

Herein, an XGBoost ML method was used to develop predictive models for bio-oil yield and solid residue from base-catalyzed lignin depolymerization reactions, achieving *R*^2^ scores of 0.80 and 0.87, respectively, for the best model in each case. Results indicate that the relations between reaction parameters such as temperature and ratio of lignin to catalyst or solvent do not follow a linear relationship and are different for bio-oil yield and solid residue yield prediction. Yields of bio-oil and solid residue may be poor metrics for reaction performance evaluation. Therefore, alternative metrics such as target chemical concentration or chemical bond concentration were suggested. Based on the contrast between the modeling and experimental work done, recommendations on how to report experimental results from lignin depolymerization were suggested, including proper characterization of lignin properties and experimental techniques, although more in-depth discussion and analysis is necessary for defining useful guidelines. Future research regarding hydrothermal lignin liquefaction (catalyzed or not) should ideally aim to take advantage of data science and ML tools to address fundamental phenomenological issues such as under what conditions can C–O–C and C–C bonds be broken and what role (beneficial or not) do certain characteristics of lignin play, such as molecular weight distribution and ash content.
